# Laparoscopic simultaneous anterograde inguinal and pelvic lymphadenectomy for penile cancer: two planses, three holes, and six steps

**DOI:** 10.3389/fsurg.2024.1344269

**Published:** 2024-05-30

**Authors:** Xian-lin Yi, Xiu-ning Li, Yu-lei Lu, Hao-yuan Lu, Yu Chen, Li-xia Zeng, Wen Qin, Yun Wu, Yong Tang

**Affiliations:** ^1^ Department of Urology, Wuming Hospital of Guangxi Medical University, Nanning, China; ^2^ Department of Urology, Maternal and Child Health Hospital of Hubei Province, Tongji Medical College, Huazhong University of Science and Technology, Wuhan, China; ^3^ Department of Urology, Guangxi Medical University Cancer Hospital, Nanning, China

**Keywords:** penile cancer, metastasis, laparoscopy, anterograde, inguinal lymphadenectomy

## Abstract

**Objective:**

To assess the feasibility, safety, and efficiency of simultaneous anterograde video laparoscopic inguinal and pelvic lymphadenectomy for penile cancer.

**Materials and methods:**

We reviewed retrospectively the records of 22 patients (44 lateral) who underwent inguinal lymph nodes dissection for penile cancer. The procedure was standardized as two planes, three holes, and six steps. Two Separate-planes: superior plane of eternal oblique aponeurosis/ / fascia lata; inferior plane of superficial camper fascia. Three holes: two artificial lateral boundary holes, the internal and external boundary holes, and the hole of oval fossa. Six steps: separate the first separate-plane; separate the second layer; separate two artificial lateral boundary holes; free great saphenous vein; separate the third hole and clean up the deep inguinal lymph nodes; pelvic lymphadenectomy.

**Results:**

A total of 22 cases were included and 9 patients underwent simultaneous pelvic lymphadenectomy. The average operation time on both sides was 7.52 ± 3.29 h, which was 0.5–1 h/side after skilled. The average amount of bleeding was 93.18 ± 50.84 ml. A total of 8 patients had postoperative complications, accounting for 36.36%, and no complications great than Clavien-Dindo class III occurred.

**Conclusion:**

This study demonstrated that the video laparoscopic simultaneous anterograde inguinal and pelvic lymphadenectomy is a feasible and safe technique. Indocyanine Green was helpful for lymph node identify.

## 1 Introduction

Penile cancer is a relatively rare tumor, but it has a very strong social stigma (1). Lymph node metastasis is the primary survival factor (1). The prognosis of patients with inguinal lymph node metastasis is poor. Inguinal and pelvic lymphadenectomy can significantly improve survival rates.

As early as 1948, Daseler et al. (2) first proposed radical inguinal lymphadenectomy. The approach has a good tumor control effect, but it is associated with great trauma and a high incidence of postoperative complications. To reduce postoperative complications, Catalona et al. (3) modified the scope of dissection and preserved the great saphenous vein in 1988. Bishoff et al. (4, 5) performed endoscopic inguinal lymphadenectomy in 2003. Tobias Machado performed video endoscopic inguinal lymphadenectomy (VEIL) in 2005 (6). VEIL has less trauma, skin complications, lymphedema, hospital stay, duration of drainage, and better lymph node yield than open operation (7).

Currently, there is no unified surgical method for inguinal lymphadenectomy (8). The reported approaches include the hypogastric subcutaneous approach, leg subcutaneous approach (VEIL-L) (9), Lateral VEIL approach (8), and the Pelvic and Inguinal Single Access (PISA) approach (10). In conventional VEIL, a trocar is placed on the thigh and above the knee joint. However, the camera can hit the knee, the operator need move to reach the camera, and the instruments may be sword fighting (8).

Therefore, we used an anterograde approach (hypogastric subcutaneous approach) to evaluate the efficacy and safety of VEIL at our institute. Some patients underwent pelvic lymphadenectomy simultaneously. In this study, we aimed to introduce the laparoscopic antegrade inguinal lymphadenectomy procedure for penile cancer and explore the effects and complications of this procedure.

## 2 Materials and methods

### 2.1 Clinical characteristics of patients

We retrospectively collected the records of 22 consecutive patients with penile cancer who underwent video endoscopic inguinal lymphadenectomy (VEIL) at Wuming Hospital and Cancer Hospital of Guangxi Medical University between January 2011 and December 2022. The inclusion criteria were as follows: (1) preoperative pathological diagnosis of penile carcinoma, stage T1b to T4. (2) complete clinical and follow-up data; (3) all patients underwent laparoscopic anterograde inguinal lymphadenectomy, including patients who underwent pelvic lymphadenectomy at the same time; and (4) with palpable inguinal lymphadenopathy and had indications for lymphadenectomy, no distant metastasis before surgery.

### 2.2 Preoperative preparation and equipment

Ultrasonography and enhanced computed tomography (CT), or magnetic resonance imaging (MRI), or PET/CT were performed to evaluate local lymph node status and distant metastasis. Pathological staging was performed based on the Tumor Node Metastasis (UICC TNM) grading system (11) (Supplementary Table S1). Anticoagulant agents were stopped one week before surgery. All patients preoperatively washed the perineum with 3% benzalkonium chloride for 3 days. All patients received general anesthesia and preoperative antibiotic therapy.

### 2.3 Surgical procedure

**Figure 1:**
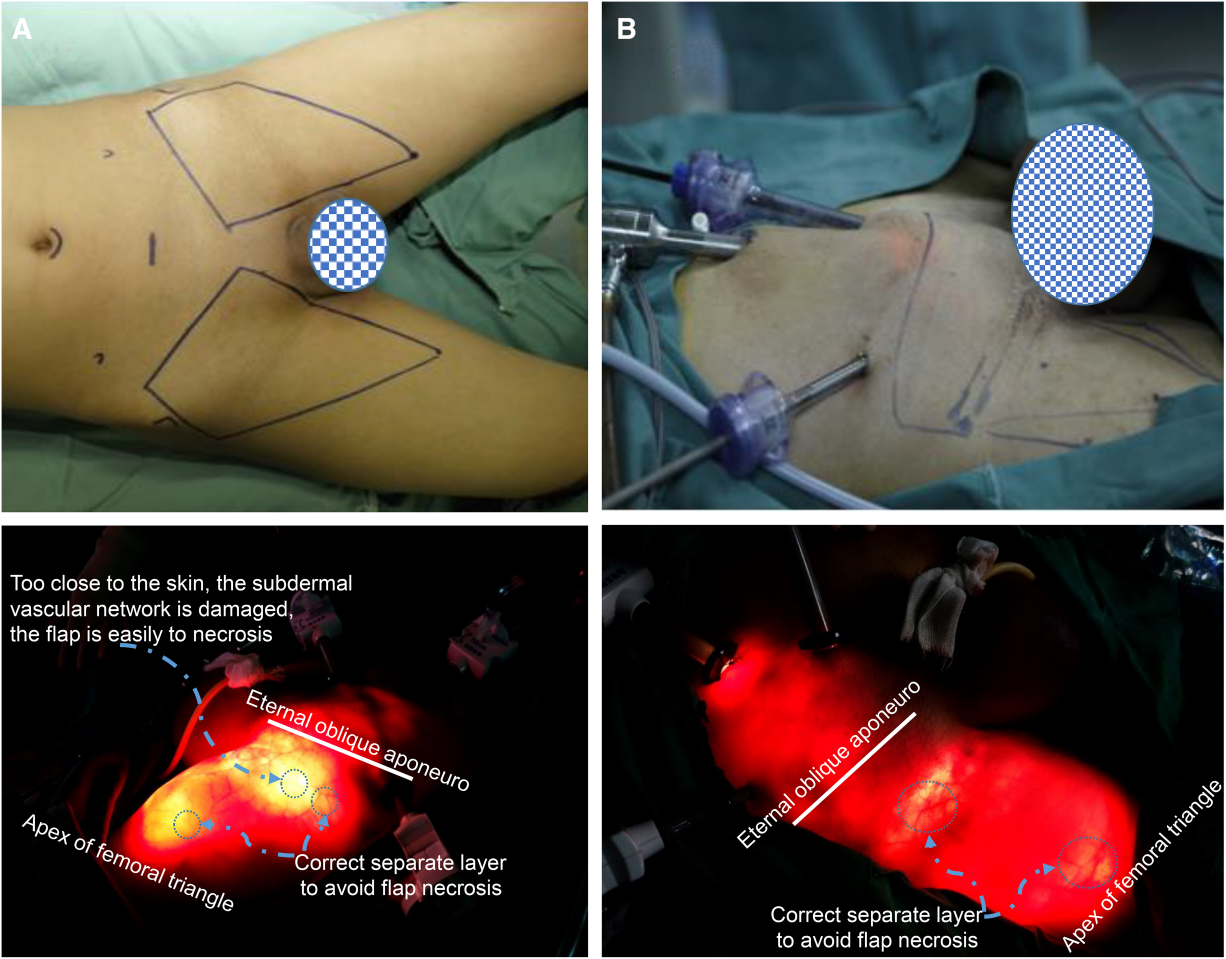
The scope of laparoscopic anterograde inguinal lymphadenectomy and the location of the trocar. (**A**) The patient was positioned in a supine position, and the two limbs were externally rotated at 30°. The scope of lymphadenectomy, 3 cm above the inguinal ligament, was the upper boundary; the inside of the sartorius muscle was the outside boundary; the side of the adductor muscle was the outer boundary; and the top of the femoral triangle was the lower boundary. (**B,A**) 10 mm trocar was placed under the umbilical margin. A 10 mm trocar was inserted at the midpoint of the connecting line between the umbilicus and pubic symphysis, and a 5 mm or 10 mm trocar was inserted into the outer 1/3 of the connecting line between the umbilicus and anterior superior iliac spine on the same side to perform lymphadenectomy.

All patients underwent laparoscopic anterograde inguinal ± pelvic lymphadenectomy performed by the same operator using the same surgical method (Supplementary Table S2). Some patients underwent total or partial penectomy and lymphadenectomy simultaneously (Supplementary Table S2). The scope of lymphadenectomy, 3 cm above the inguinal ligament, was the upper boundary; the inside of the sartorius muscle was the outside boundary; the side of the adductor muscle was the outer boundary; and the top of the femoral triangle was the lower boundary (Figure 1).

#### 2.3.1 Position and trocar placement

The patient was placed in a supine position, and the two limbs were externally rotated at 30°.

For bilateral inguinal lymphadenectomy, four trocars were placed in the lower abdomen. An arc incision was placed under the umbilical margin, a 10 mm trocar was placed for mirror observation; A 10 mm trocar was inserted at the midpoint of the connecting line between the umbilicus and the pubic symphysis, which was the operative passageway. This operative passageway and the operative passageway were shared if lymphadenectomy was performed on both sides. A 5 mm or 10 mm trocar was inserted into the outer 1/3 of the connecting line between the umbilicus and anterior superior iliac spine on the same side to perform lymphadenectomy. These trocar positions can be used simultaneously for pelvic lymph node dissection (Figure 1).

#### 2.3.2 This anterograde VEIL approach can be simplified as two planes, three holes, and six steps

##### 2.3.2.1 Two separate-planes

Superior plane of the eternal oblique aponeuro/fascia lata; inferior plane of the superficial camper fascia. This anterograde approach should separate the external oblique fascia and the deep interposition plane of the camper fascia, namely, the Scarpa fascia plane. After crossing the inguinal ligament, a deep interposition plane was observed between the superficial femoral fascia and fascia lata, which was the first separate plane. The first plane was then separated from the superficial layer to find the plane between the superficial and deep layers of the camp fascia, which was the second separate plane.

##### 2.3.2.2 Three holes: two artificial lateral boundary holes, the lateral adductor hole and the medial sartorius hole, and the hole of the oval fossa

In the first separation plane, two artificial channels were separated along the outside of the adductor muscle and inside of the sartorius muscle, which were called internal and external boundary holes. The two holes converge at the top of the femoral triangle to reach the lower boundary. It was separated downward along the second layer, and the boundary was the same as that of the first separate plane. The great saphenous vein was then separated and naked (the great saphenous vein was located at approximately one transverse finger below the middle and inner 1/3 intersection of the inguinal ligament). Most branches of the great saphenous vein were ligated and the main trunk was retained. If possible, the main trunk and the medial femoral vein were maintained.

So far, the lymphoid adipose tissue between the first and second separate**-**planes has been removed and superficial inguinal lymphadenectomy has been completed.

Finally, the deep inguinal lymph nodes were removed. The cribriform fascia on the surface of the oval fossa was then opened. The deep lymph nodes were mostly located above the confluence of the great saphenous vein with the femoral vein and around the upper segment of the femoral artery and vein. The femoral sheath was opened and lymphoid adipose tissue was cleaned around the femoral artery and vein.

#### 2.3.3 Six steps

##### 2.3.3.1 Separate the first separate-plane (superior plane of external oblique aponeuro/fascia lata)

**Figure 2:**
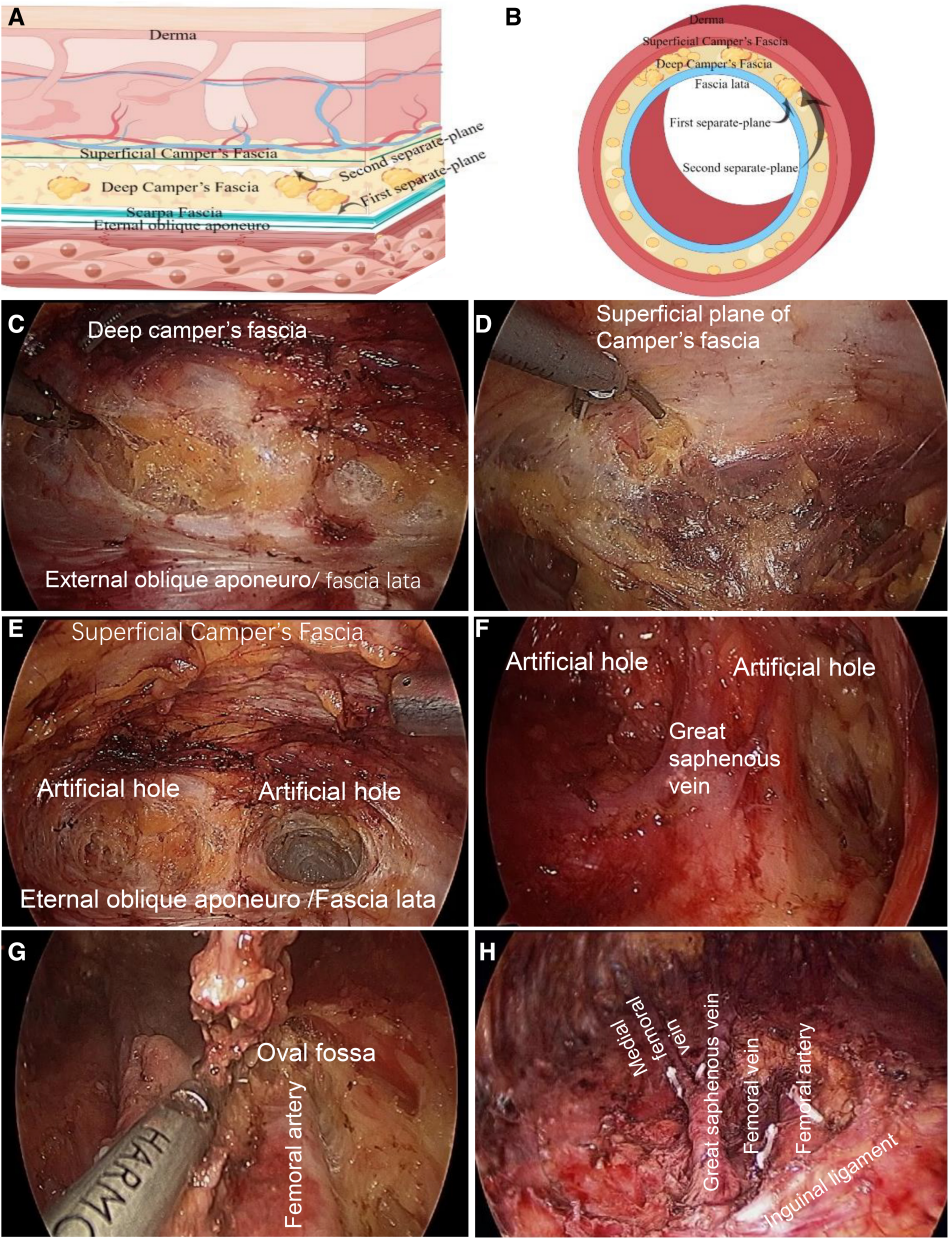
Two planes and three holes of superficial and deep inguinal lymphadenectomy. (**A**) The superficial anatomical structure of the cephalic abdomen above the inguinal ligament, the first and second separate-planes are shown in a transverse position. (**B**) The first and second separate-planes of the superficial anatomy of the thigh across the inguinal ligament are shown in the transverse axial. Find the superficial and deep layer of camper fascia, which is the second separate-plane. The separation order of the first and second separate-planes can be reversed. Surgeons can advise starting at the second separate-plane at first. (Some materials of Figure A and B from figdraw. https://www.figdraw.com/, ID:YPOIUa1aaa). (**C**) Firstly, in the cephalic part of the inguinal ligament, the ultrasonic scalpel was used to separate the first separate plane along the deep plane between the external oblique muscle fascia and camper fascia, that is, the Scarpa fascia plane; After passing through the inguinal ligament, it is the plane between the deep layer of superficial femoral fascia and fascia lata. (**D**) Separate from the first separate-plane to the shallow layer, and find the shallow and deep plane of camper fascia, which is the second separate-plane. (**E**) Two artificial lateral boundary holes. Two artificial channels were separated along the outside of the adductor muscle and inside of the sartorius muscle on the first separate plane, which were the inner and outer boundary holes. The two holes converge at the femoral triangle. Thus far, the two sides of the sweeping range and lower boundary have been separated. (**F**) The second separate-plane was separated after the first separate-plane was free, and artificial border holes on both sides were constructed. Subsequently, the great saphenous vein was free, the branches were disconnected, the trunk was kept, and the superficial inguinal lymph nodes were completely removed. (**G**), Clean the deep inguinal lymph nodes. The cribriform fascia was opened on the oval fossa surface. In the deep group, the lymph nodes were mostly located above the confluence of the great saphenous vein with the femoral vein and around the upper segment of the femoral artery and vein. The lymphoid adipose tissue was cleaned, the femoral sheath was opened, and the lymphoid adipose tissue around the femoral artery and vein was cleaned. (**H**) Inguinal lymph node dissection is performed.

Separate the external oblique fascia and the deep interposition plane of the camper fascia, that is, the Scarpa fascia layer. After crossing the inguinal ligament, the deep interposition plane between the superficial femoral fascia and fascia lata is the first separate plane (Figures 2A–C).

##### 2.3.3.2 Separate the second layer (superficial inferior plane of camper fascia)

Separated the first separate plane from the shallow layer and found the plane between the shallow and deep layers of the camper fascia, which is the second separate plane (Figures 2A,B,D).

##### 2.3.3.3 Separate two artificial lateral boundary holes

From the first separate-plane, it separates downward along the outside of the adductor muscle and the inside of the sartorius muscle, and separates two artificial channels, which are called internal and external boundary holes. The two holes at the top of the femoral triangle converged to form a lower separated boundary. The separated boundary of the second plane was the same as that of the first plane. The order of separation of the first and second separate planes can be reversed. Surgeons can advise starting with the second separate plane (Figures 2E,F).

##### 2.3.3.4 Free great saphenous vein

The great saphenous vein was separated downward at the first separate-plane, and the great saphenous vein (the great saphenous vein was located at approximately one transverse finger below the middle and inner 1/3 intersection of the inguinal ligament). After finding the great saphenous vein, it is separated downward and naked to the top of the femoral triangle, disconnects the branches of the great saphenous vein, and retains the main trunk of the great saphenous vein and its branch, the medial femoral vein. So far, the lymphoid adipose tissue between the first and second layers has been removed and superficial inguinal lymph node dissection has been completed.

##### 2.3.3.5 Separate the third hole and clean up the deep inguinal lymph nodes

The cribriform fascia was opened on the surface of the oval fossa. The deep inguinal lymph nodes were mostly located above the confluence of the great saphenous vein with the femoral vein and around the upper segment of the femoral artery and vein. Clean up lymphoid adipose tissue The femoral sheath was opened and the lymphoid adipose tissue around the femoral artery and vein was cleaned (Figures 2G,H).

##### 2.3.3.6 Pelvic lymphadenectomy

When frozen or routine pathology showed more than two positive inguinal lymph nodes, bilateral pelvic lymphadenectomy was performed simultaneously. The same inguinal lymphadenectomy incision was used for pelvic lymphadenectomy.

### 2.4 Postoperative management

A local compression bandage was applied to each inguinal area in all patients. The drain was maintained until the drainage volume was less than 20 ml within 24 h.

### 2.5 Statistical analysis

SPSS software (version 22.0, USA) was used for data analysis. All quantitative values are expressed as the median ± SD. The description of the counting data is expressed by the sample size and composition ratio, *n* (%). Comparisons of each group were performed using the chi-square test. Statistical significance was set at *P* < 0.05.

## 3 Results

A total of 22 cases (44 sides) were included in this study. Their ages ranged from 25 to 66 years, with an average of 49.55 ± 12.54 years. The clinical characteristics and pathological stages are shown in Supplementary Table S1.

### 3.1 Operative time

A total of 44 limbs were successfully treated in 22 surgical procedures. The average operative time was 7.52 ± 3.29 h, the longest was 12.8 h, and the shortest was 3.0 h, including the time of total or partial penectomy and pelvic lymphadenectomy. The duration of bilateral inguinal lymphadenectomy alone was not calculated separately. In the latter patients, laparoscopic anterograde inguinal lymphadenectomy was performed 0.5–1 h/side.

### 3.2 Intraoperative bleeding and blood transfusion

The average amount of bleeding was 93.18 ± 50.84 ml. During the operation, eight patients received blood transfusions, all of which were plasma transfusions, no red blood cell transfusions, and no platelet transfusions (Supplementary Table S2).

### 3.3 Postoperative inguinal lymphadenectomy

Twelve cases with inguinal lymph node metastasis were confirmed by pathology, including nine cases of unilateral and three cases of bilateral metastasis. Extranodal lymph node invasion occurred in four cases. The average number of lymph nodes in each patient was 28.95 ± 15.98, while the number of positive lymph nodes was 2.05 ± 4.59 (Supplementary Table S2).

### 3.4 The duration of drainage

The duration of drainage was 18.55 ± 17.06 days (Supplementary Table S2).

### 3.5 Postoperative complications

A total of 8 patients had postoperative complications, accounting for 36.36%. However, no serious complications and or complications greater than Clavien-Dindo class III occurred.

Postoperative wound infection occurred in 7 cases, flap necrosis in 2, lymphorrhagia in 3, and lymphatic cyst in 1. No venous thrombosis, edema, seroma, or paresthesia was observed (Supplementary Table S4). All two cases of flap necrosis were small-scale necrosis, which was cured by conservative and symptomatic treatment without skin grafting.

### 3.6 Comparison between contemporaneous and asynchronous inguinal and pelvic lymphadenectomy group

When compared to the asynchronous inguinal and pelvic lymphadenectomy groups, the total number of lymph nodes was different in the contemporaneous group. However, there was no difference in the number of positive lymph nodes and in the length of stay, bleeding volume, and postoperative complications (Supplementary Tables S3, S4).

### 3.7 Fluorescent and nano-carbon staining were helpful for intraoperative identify lymph nodes

**Figure 3:**
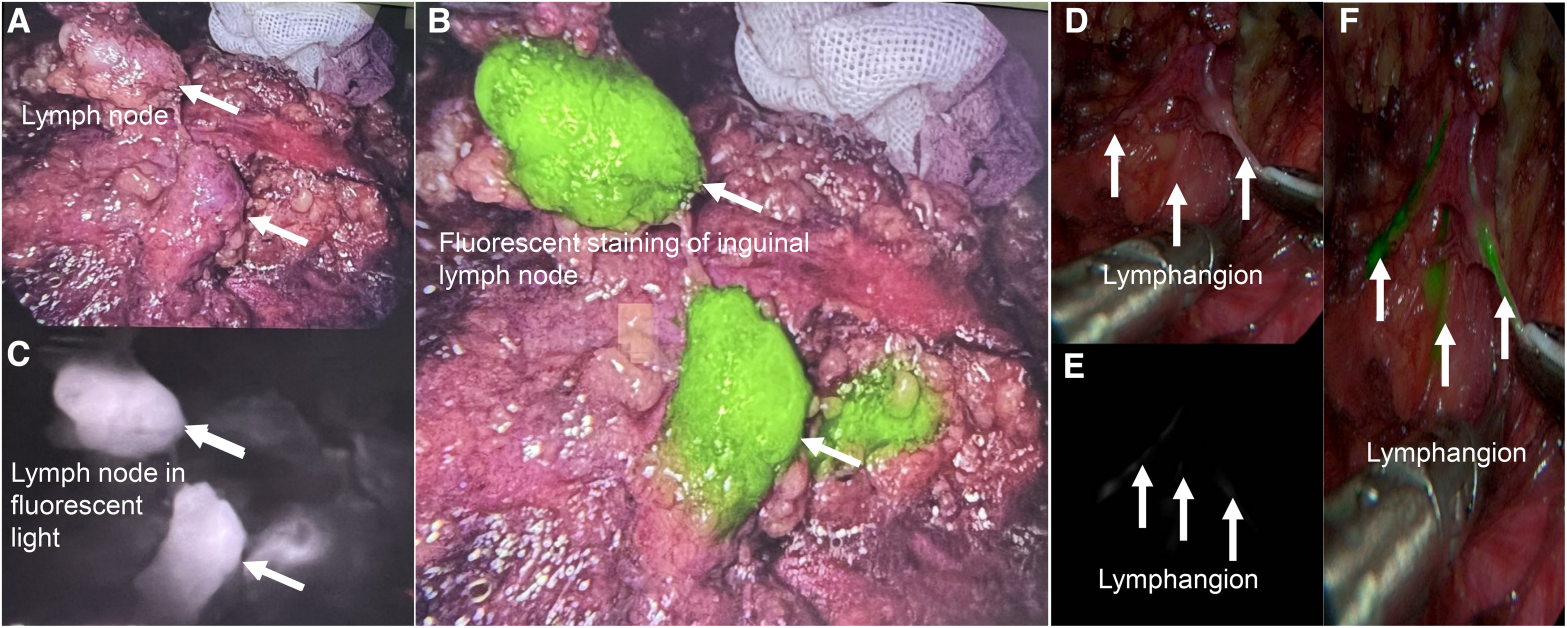
Fluorescent staining was useful for intraoperative lymph node identify. (**A**) Imaging of inguinal lymph nodes without fluorescent staining *in vivo*. (**B**), The same lymph node in the black and white fluorescence mode. (**C**), The same lymph node with fluorescent staining in colorful mode. (**D–F**), Lymphangion with fluorescent staining.

To better identify lymph nodes, we used Indocyanine Green (Figure 3) and Nano-carbon (Supplementary Figure S1) to identify them. (Figure 3), both of which were helpful in surgery.

## 4 Discussion

Classical radical inguinal lymphadenectomy is the most effective treatment for inguinal lymph node metastasis in penile cancer. However, this procedure is highly traumatic. The incidence of major complications in inguinal lymphadenectomy can reach 40%–55% (1). It has even been reported that the incidence of postoperative complications can reach to 85% (12). In 2011, we first reported contemporaneous laparoscopic anterograde inguinal and pelvic lymphadenectomy for penile cancer in China (13). After the operator is skilled, the time of unilateral lymphadenectomy is 0.5–1 h, which is shorter than that of open dissection. The incidence of postoperative complications in our series was 36.8%, which was lower than that in the previous open surgery (68.4%), and no serious complications occurred in our series. The complications of incision infection was showed in six cases, flap necrosis in two cases, lymphorrhagia in two cases, and lymphatic cyst in one case. However, the degree of incision infection was mild. And the scope of the flap necrosis was small and self-healed without surgical intervention.

Inguinal lymphadenectomy is accompanied by a high risk of complications such as skin necrosis, seroma, local edema, infection, lymphocele, lymphorrhea, and thrombophlebitis (1). To reduce the incidence of complications, Catalona et al. (3) proposed improved inguinal lymphadenectomy in 1988. The scope of the dissection was narrower than that of traditional radical surgery. This procedure is more suitable for patients with negative inguinal lymph nodes. A smaller incision was made, the great saphenous vein was reserved, and the lymph nodes near the femoral artery and deep oval fossa were not cleaned during the procedure. The tumor control effect was equivalent to that of the traditional radical inguinal lymphadenectomy. The incidence of postoperative complications is lower than that of traditional radical inguinal lymphadenectomy, but the incidence can still reach 50% (14, 15).

Compared with open surgery, laparoscopic anterograde inguinal lymphadenectomy can avoid long bilateral incisions and achieve the goal of minimally invasive surgery. Compared with retrograde inguinal lymphadenectomy via femoral puncture, anterograde VEIL avoided instruments fighting (8). In addition, the number of puncture holes was lower in our anterograde VEIL. Only four puncture holes were needed to complete bilateral inguinal lymphadenectomy, while retrograde dissection required six puncture holes on both sides. After dissection of the lymph node on one side with the same trocar, we continued to dissect that on the other side and used the existing incision to perform pelvic lymphadenectomy at the same time. There is no need to replace trocars, as in the retrograde approach. Our anterograde VEIL is more convenient and reduces the operative time and trauma after the surgeons are familiar with this anterograde VEIL procedures.

The key points of laparoscopic anterograde inguinal lymphadenectomy can be summarized as two sides, three holes, three tubes, and six steps. The superficial adipose tissue of the camper fascia is preserved and the blood supply of the flap is protected, which is helpful in preventing flap necrosis, nonunion, and infection after the operation. Three tubes were the femoral artery, femoral vein, and great saphenous vein.

Kumar et al. (16) reported that the average dissection of lymph nodes was 9.36, while Delman et al. (17) was 14. Our results showed that the average number of lymph nodes removed during inguinal lymphadenectomy for penile cancer was 21.79 ± 10.72. Nano carbon and indocyanine green injection can help identify these lymph nodes.

The average total operation time of the patients in the current study was 8.13 ± 3.12 h, including partial or total penectomy and pelvic lymphadenectomy. Because the time of laparoscopic anterograde inguinal lymphadenectomy was not calculated independently, the average operation time for inguinal lymphadenectomy was difficult to determine. However, with the maturity of the technology, the operation time gradually shortened, and the unilateral dissection time of several patients in the later skilled operator was 0.5–1 h, which was equivalent to some similar studies (18, 19). The most time-consuming step is separating the great saphenous veins. In the initial cases, we tried to preserve all the branches of the great saphenous vein, although they were successfully preserved, but the longest operating time was 12.8 h. Subsequently, we found that it was not necessary to keep all of the branches of the great saphenous vein, and the operating time was greatly shortened. Some surgeons suggest maintaining only the main trunk of the great saphenous vein. However, a recent report suggested that preserving parts of the superficial branches of the great saphenous vein decreases the incidence of postoperative complications (20). Our experience was to maintain the main trunk lymphadenectomy and its branch, the medial femoral vein, unless there was bulky disease that the myocutaneous flap and skin graft was necessary. Our results confirmed that this approach has less impact on the operative time and operative difficulty and can better reduce the occurrence of lower limb edema.

This study had some limitations. At first, the number of cases included was small; therefore, it is still necessary to continue collecting cases. The follow-up time was short, and the survival analysis of postoperative patients was not fit. Second, the duration of drainage was long, because some rural patients with lymphorrhagia were unwilling to frequently return to the hospital for syringe suction. Finally, 10/22 patients with pN0 may raise some concern regarding the indication used for a negative staging procedure, although most of these patients showed positive preoperative imaging results with palpable inguinal lymphadenopathy and underwent surgery according to their desire.

## 5 Conclusion

In conclusion, a novel technique of antegrade lymph node dissection in penile cancer is proposed in which the pelvic and inguinal areas can be approached through the same working channels in several simplified steps. The key points to be grasped during the operation are the two sides, three holes, three tubes, and six steps.

The main trunk of the great saphenous vein and its branch medial femoral vein are technically feasible and can help reduce the occurrence of lower limb edema.

## Data Availability

The original contributions presented in the study are included in the article/**Supplementary Material**, further inquiries can be directed to the corresponding author.

## Supplementary material

Supplementary Figure S1 Nano-carbon stain of penile and inguinal pelvic lymph nodes. (**A**) Excised penile with black staining by Nano-carbon. (**B**) Excised inguinal lymph nodes stained black.

Supplementary Figure S2 Fluorescent staining was useful for intraoperative lymph node identify both *in vivo* and vitro. (**A**) Excised inguinal lymph node after fluorescent staining *in vitro*. (**B**) Excised inguinal lymph node in black and white fluorescence mode. (**C**) Excised inguinal lymph node in colorful fluorescence mode.
