# Supplementary material for: Laparoscopic simultaneous anterograde inguinal and pelvic lymphadenectomy for penile cancer: two planses, three holes, and six steps
Source: Front Surg. 2024 May 30;11:1344269. doi: 10.3389/fsurg.2024.1344269 (PMC11169933; doi:10.3389/fsurg.2024.1344269)
Supplement: Supplementary file 1 [file Table1.docx]

**Supplementary Table 1.** **Clinical characteristics of 22 patients undergoing laparoscopic inguinal lymphadenectomy**

| **Variable** | **N** |
| --- | --- |
| Cases(N) | 22（44 lateral） |
| **T stage** |  |
| T1b | 4 |
| T2 | 11 |
| T3 | 4 |
| T4 | 1 |
| Tx | 2 |
| **pN stage** |  |
| pN0 | 10 |
| pN1 | 5 |
| pN2 | 2 |
| pN3  Pathological grade  well differentiated moderately differentiated poorly differentiated | 5  12  3  7 |
|  |  |
|  |  |
|  |  |
