# Supplementary material for: Laparoscopic simultaneous anterograde inguinal and pelvic lymphadenectomy for penile cancer: two planses, three holes, and six steps
Source: Front Surg. 2024 May 30;11:1344269. doi: 10.3389/fsurg.2024.1344269 (PMC11169933; doi:10.3389/fsurg.2024.1344269)
Supplement: Supplementary file 2 [file Table2.docx]

**Table 1.** **Clinical characteristics of operation and postoperative complications**

| **Variable** | **Account** |
| --- | --- |
| **Surgical method** | N（%） |
| Bilateral inguinal lymphadenectomy alone | 5(22.73) |
| Bilateral inguinal + pelvic lymphadenectomy | 2(9.10) |
| Total or partial penectomy + bilateral inguinal and pelvic lymphadenectomy | 7(31.81) |
| Total or partial penectomy + bilateral inguinal lymphadenectomy | 8(36.36) |
| **Intraoperative blood loss and transfusion** |  |
| Total Blood loss ( Volume,ml) | 93.18±50.84 |
| Intraoperative transfusion of red blood cells | N（%） |
| yes | 0/22(0.00) |
| no | 22/22(100.00) |
| Intraoperative plasma transfusion | N（%） |
| yes | 8/22(36.36) |
| no | 14/22(63.64) |
| Intraoperative platelet transfusion | N（%） |
| yes | 0/22(0.00) |
| no | 22/22(100.00) |
|  |  |
| **Results of lymphadenectomy** | N（%） |
| Location of positive lymph nodes | N（%） |
| unilateral | **9/12(75.00)** |
| bilateral | **3/12(25.00)** |
| Extranodal invasion of lymph nodes |  |
| yes | 4/22(18.18) |
| no | 18/22(81.82) |
| Total number of lymphadenectomy | 28.95±15.98 |
| Number of positive lymph nodes | 2.05±4.59 |
| **Time of drainage tube stay(days)** | **18.55±17.06** |
|  |  |
| **Postoperative complications** |  |
| **Necrosis of skin flap (a small area)** | N（%） |
| yes | 2/22(9.10) |
| no | 20/22(90.90) |
| **Subcutaneous infection** | N（%） |
| yes | 7/22(31.82) |
| no | 15/22(68.18) |
| **Lymphorrhagia** | N（%） |
| yes | 3/22(13.64) |
| no | 19/22(83.36) |

N, cases
