# Supplementary material for: Laparoscopic simultaneous anterograde inguinal and pelvic lymphadenectomy for penile cancer: two planses, three holes, and six steps
Source: Front Surg. 2024 May 30;11:1344269. doi: 10.3389/fsurg.2024.1344269 (PMC11169933; doi:10.3389/fsurg.2024.1344269)
Supplement: Supplementary file 3 [file Table3.docx]

**Supplementary Table 3** Comparison of inguinal lymphadenectomy alone and simultaneous inguinal + pelvic lymphadenectomy

| **Variable** | **N=13** | **N=9** | ***P* Value** |
| --- | --- | --- | --- |
| Total number of lymph nodes | 21.15±11.24 | 40.22±15.46 | 0.003 |
| Positive lymph nodes | 0.92±1.93 | 3.67±6.69 | 0.174 |
| ospital stay days | 33.08±12.39 | 32.56±9.70 | 0.917 |
| Total Blood loss ( Volume,ml) | 76.15±43.12 | 117.78±53.33 | 0.057 |
| Subcutaneous infection |  |  | 0.629 |
| no | 10/13(76.92%) | 5/9(55.56%) |  |
| yes | 3/13(23.08%) | 4/9(44.44%) |  |
| Necrosis of skin flap |  |  | 0.940 |
| no | 12/13(92.31%) | 8/9(88.89%) |  |
| yes | 1/13(7.69%) | 1/9(11.11%) |  |
| Lymphorrhagia |  |  | 0.829 |
| no | 11/13(84.62%) | 8/9(88.89%) |  |
| yes | 2/13(15.38%) | 1/9(11.11%) |  |
