# Supplementary material for: Laparoscopic simultaneous anterograde inguinal and pelvic lymphadenectomy for penile cancer: two planses, three holes, and six steps
Source: Front Surg. 2024 May 30;11:1344269. doi: 10.3389/fsurg.2024.1344269 (PMC11169933; doi:10.3389/fsurg.2024.1344269)
Supplement: Supplementary file 4 [file Table4.docx]

**Supplementary Table 4** The number of inguinal and pelvic lymph nodes

| **Variable** | Total number | Mean ± standard deviation | *P* Value |
| --- | --- | --- | --- |
| Total number of inguinal lymph nodes | 409 | 22.722±9.828 | 0.111 |
| Total pelvic lymph nodes | 144 | 16.00±10.283 |  |
| Inguinal positive lymph nodes | 44 | 2.44±4.768 | 1.59 |
| Pelvic positive lymph nodes | 1 | 0.111±0.333 |  |
