# Supplementary figures and images for: Laparoscopic simultaneous anterograde inguinal and pelvic lymphadenectomy for penile cancer: two planses, three holes, and six steps
Source: Front Surg. 2024 May 30;11:1344269. doi: 10.3389/fsurg.2024.1344269 (PMC11169933; doi:10.3389/fsurg.2024.1344269)

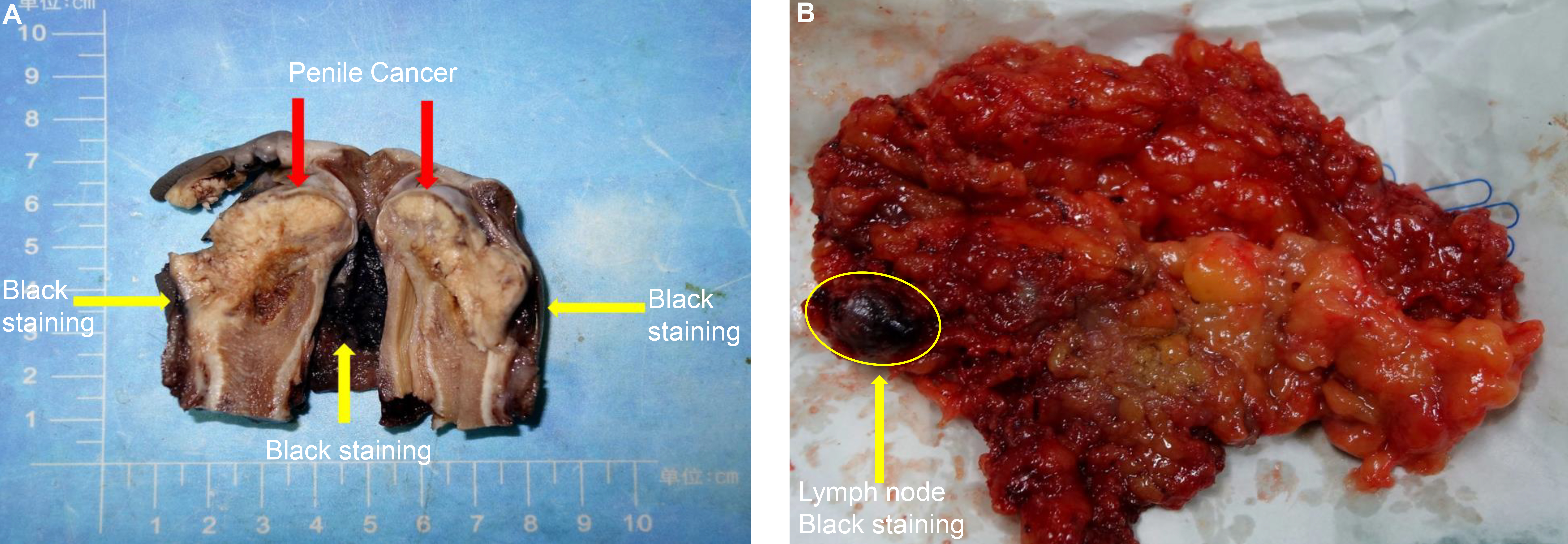

Supplement: Supplementary Figure S1 — Nano-carbon stain of penile and inguinal pelvic lymph nodes. (A) Excised penile with black staining by Nano-carbon. (B) Excised inguinal lymph nodes stained black. [file Image1.jpeg]

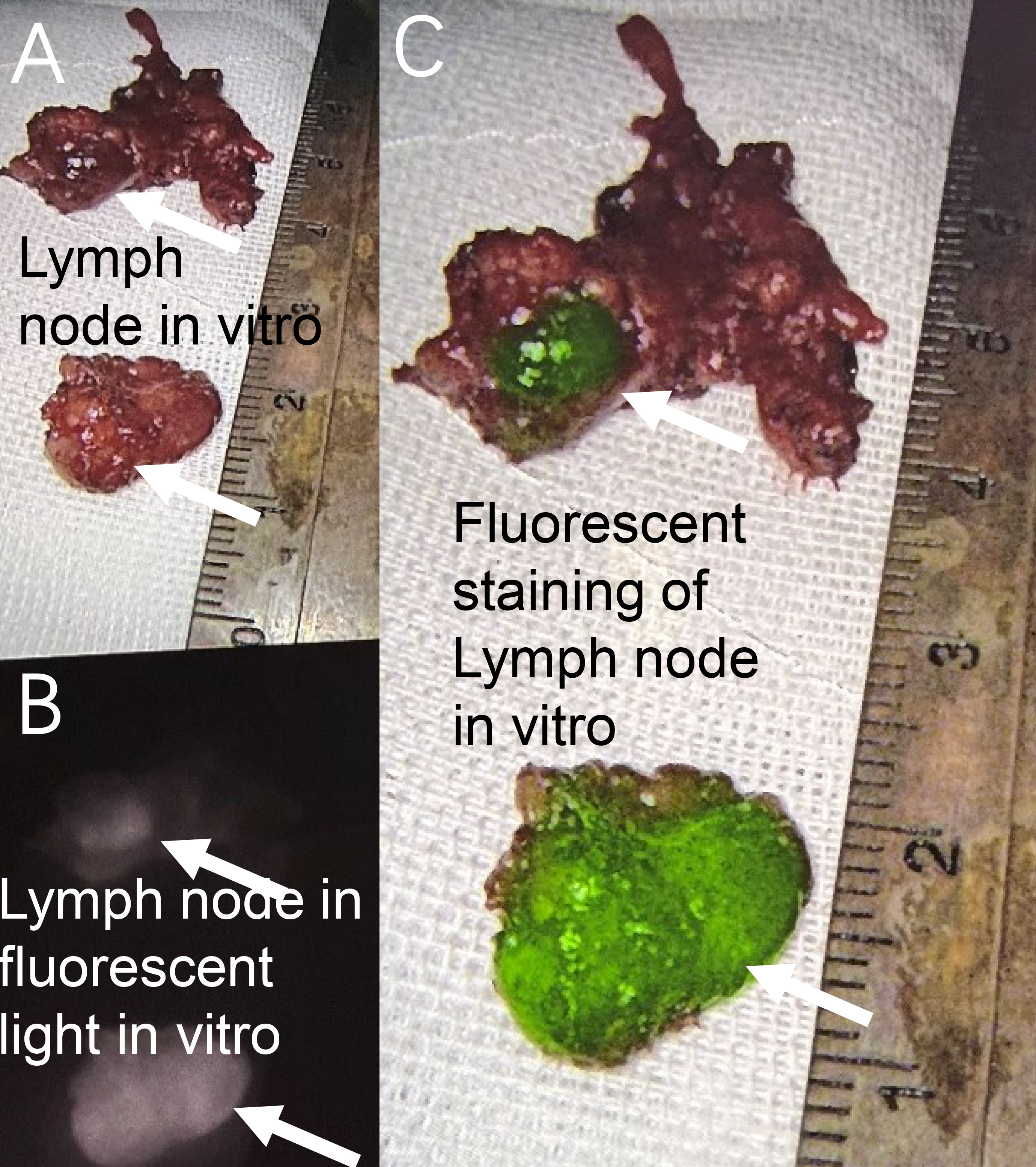

Supplement: Supplementary Figure S2 — Fluorescent staining was useful for intraoperative lymph node identify both in vivo and vitro. (A) Excised inguinal lymph node after fluorescent staining in vitro. (B) Excised inguinal lymph node in black and white fluorescence mode. (C) Excised inguinal lymph node in colorful fluorescence mode. [file Image2.jpeg]
